# Skin hydration: interplay between molecular dynamics, structure and water uptake in the stratum corneum

**DOI:** 10.1038/s41598-017-15921-5

**Published:** 2017-11-16

**Authors:** Enamul Haque Mojumdar, Quoc Dat Pham, Daniel Topgaard, Emma Sparr

**Affiliations:** Division of Physical Chemistry, Center for Chemistry and Chemical Engineering, Lund University, P.O. Box 124, SE-22100 Lund, Sweden

## Abstract

Hydration is a key aspect of the skin that influences its physical and mechanical properties. Here, we investigate the interplay between molecular and macroscopic properties of the outer skin layer – the stratum corneum (SC) and how this varies with hydration. It is shown that hydration leads to changes in the molecular arrangement of the peptides in the keratin filaments as well as dynamics of C-H bond reorientation of amino acids in the protruding terminals of keratin protein within the SC. The changes in molecular structure and dynamics occur at a threshold hydration corresponding to ca. 85% relative humidity (RH). The abrupt changes in SC molecular properties coincide with changes in SC macroscopic swelling properties as well as mechanical properties in the SC. The flexible terminals at the solid keratin filaments can be compared to flexible polymer brushes in colloidal systems, creating long-range repulsion and extensive swelling in water. We further show that the addition of urea to the SC at reduced RH leads to similar molecular and macroscopic responses as the increase in RH for SC without urea. The findings provide new molecular insights to deepen the understanding of how intermediate filament organization responds to changes in the surrounding environment.

## Introduction

**Figure 1:**
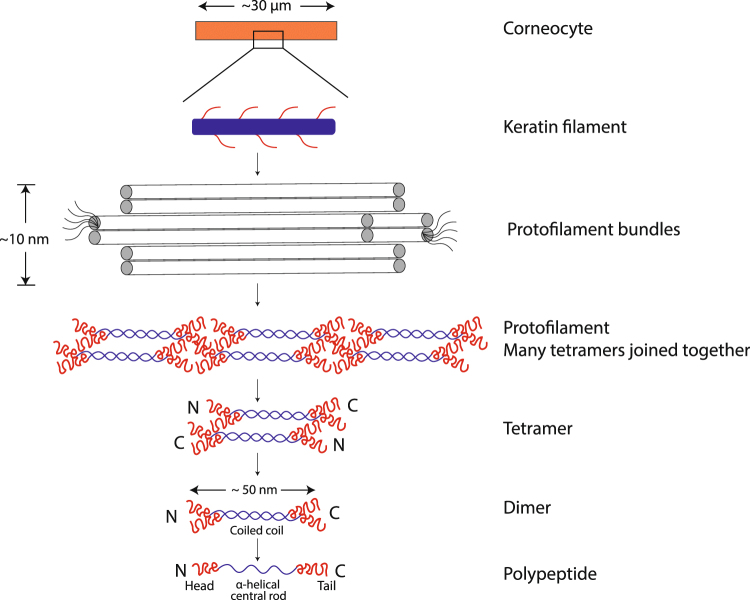
Schematic of corneocyte structural organization. Corneocytes are flattened anucleated cells with ∼30–40 μm in diameter and consist of keratin filaments^6,74,75^. The filament can be envisaged as a solid rod (blue) with protruding terminals (red) and consists of bundles of protofilaments^8–11^. The primary building block of the protofilament is a polypeptide chain that has an *α*-helical central rod with flanking N- and C-terminals. The polypeptide chain is intertwined with another keratin monomer in a parallel arrangement to form a coiled coil heterodimer of which one chain is acidic (type I) and the other is basic- neutral (type II)^9^. Two such heterodimers are then associated in an antiparallel and staggered conformation making a tetramer. The tetramers subsequently aggregate in an end-to-end fashion forming a protofilament.

The skin is a large interfacial film separating the human body and the outside environment. The outermost layer of the skin epidermis – the stratum corneum (SC) is responsible for the skin barrier function^1,2^. The healthy SC is a versatile material that combines the functional property of being an effective transport barrier, and material properties of being soft, strong and pliable to tolerate deformation from physical strain and stress. The SC is also a responding material, and its properties can be altered by changes in the skin environment^3–5^. Taken together, SC fulfills several essentially different requirements, and its special material properties can be related to the organization and dynamics of its molecular components. The SC is ca. 10–15 μm thick and consists of anucleated dead cells – corneocytes – that are filled with keratin filaments and wrapped by cornified envelope^6,7^. The keratin filaments have a rigid core with protruding terminals and they consist of bundles of protofilaments (Fig. 1)^8–11^. They have been classified as “intermediate filaments” due to their size range of ∼10–15 nm, which is intermediate compared with to the cytoskeletal actin filaments (∼6 nm) and microtubules (∼24 nm)^12,13^. Intermediate filaments are ubiquitously present in the skin, hair and nail where it acts as a mechanical scaffold. Filament structures of similar size are also found in the neurofilaments in neuron cell^12–14^. The corneocytes are embedded in a multi-lamellar lipid matrix in a fashion often described as ‘brick and mortar’ structure^15^. In ambient conditions, the vast majority of the SC lipid and protein components exist in a solid state^16–18^, which is different from most other biological membranes. Still, the presence of a small fraction of fluid components can have huge impact on the SC macroscopic material properties^5,19^.

The keratin-filled corneocytes constitute ca. 85% of the total weight of dry SC^20^, and they have been associated with SC mechanical properties. Overall, the corneocytes have a polar interior, and they may be obstacles for the diffusive transport of hydrophobic molecules, while they may provide an additional transport route for more polar compounds, for example, water. When the skin is exposed to a humid environment, the corneocytes take up substantial amounts of water^21^, which is apparent as swelling of the skin after, for example, taking a bath. Corneocytes can swell roughly 50% in height when fully hydrated^22^. The swelling of corneocytes is not uniform in all directions, and they swell more in the vertical direction compared to the parallel direction with respect to the skin surface^21,23^. The SC lipids are also affected by hydration by melting of a small portion of the lipid hydrocarbon chains^24,25^. Hydration as well leads to an increase in SC permeability at high relative humidity (RH)^26^, which can be associated with the presence of more mobile/fluid SC lipid and protein components^4,27^. This is taken advantage of in dermal and transdermal drug delivery, then called “occlusion”^28^. In occlusive condition, the skin penetration of chemicals beneath, for example, a skin patch or film of cream can be enhanced^29,30^.

The mechanical properties of the skin in terms of strength and elasticity can be affected by hydration^5,31,32^. Previous studies have reported that the water content in SC is indeed the primary factor that governs the SC flexibility^32,33^. A distinct brittle-to-supple transition has been observed in the SC sample when the moisture content was elevated to higher RH^31,34–36^. When hydrated SC, water is primarily taken up by the corneocytes. It has been hypothesized that the corneocytes control SC viscoelastic properties through the plasticization of the keratin filament macromolecules by water^34^. At low RH values, the keratin filaments are present in rigid state^24^, whereas recent studies have shown that there is a change in the molecular mobility of certain amino acids in the keratin filament upon hydration^24^. It is highly likely that these molecular changes in the keratin filaments strongly impact its swelling and mechanical properties, and this interplay is investigated in the present study. The molecular properties of the keratin can also be altered by the addition of other small polar molecules, e.g. urea and glycerol^31,37^. These molecules are naturally present in skin as part of the so-called “Natural Moisturizing Factor” (NMF), and they are commonly used in skin care products as “humectants”. Although the terminology is a bit misleading, the main function of these compounds in SC is not to increase the water content, but rather to replace water in dehydrated conditions and thereby retain fluidity in SC lipid and protein components^37^.

In the present study, we aim at deepened understanding of the nature of hydration-induced changes in keratin filaments inside the corneocytes, and of how these molecular changes can lead to alterations in macroscopic material properties. The effect of hydration on molecular organization and dynamics is also compared to the effect of adding a so-called humectant molecule commonly used in moisturizing skin care formulations. We here present data for homogenous samples, which can be used to make predictions of the effects of water and humectant at different depth in SC. The samples investigated are composed of extracted isolated corneocytes or intact SC at different hydration conditions as determined by the RH of the surrounding air. We used a multi-technique approach, including wide angle X-ray diffraction (WAXD), Fourier transform infrared spectroscopy (FTIR), polarization transfer solid state NMR (PT ssNMR)^38^ and sorption microbalance. Based on the combination of results obtained from these techniques, we can correlate observed changes in SC macroscopic properties to molecular effects in terms of dynamics, structure and conformation, thereby deepening our understanding of SC hydration.

## Materials and Methods

### Chemicals

Bovine pancreas trypsin (type III), urea, chloroform, methanol and deuterated water were purchased from Sigma-Aldrich Chemie GmbH (Schnelldorf, Germany). NaCl, Na_2_HPO_4_.2H_2_O, KH_2_PO_4_ and KNO_3_ were purchased from Merck. Water used to hydrate the SC and corneocyte samples and to prepare phosphate buffered saline (PBS) was of Millipore quality produced by MilliQ water filtration system with a resistivity of 18 MΩ·cm at 25 °C.

### SC isolation

Pig ears were collected from a local slaughterhouse and stored at −80 °C until further used. Prior to dermatoming, the pig ears were thawed and rinsed in cold tap water. The hairs were shaved using a trimmer. The inner side of the pig ears was dermatomed (TCM 3000 BL, Nouvag) to a thickness of ca. 500 μm into small slices. The slices were placed on filter papers soaked in trypsin solution (0.2 wt% trypsin in MilliQ) and kept at 4 °C for about 20 h. The SC sheets were peeled off from the remaining epidermis using forceps and washed further in MilliQ five times to get rid off all the trypsin. The SC sheets were dried under vacuum in a desiccator and stored at −20 °C for subsequent use.

### Corneocyte extraction

Corneocytes were extracted from the SC using the procedure described in^39^. Briefly, the SC sheets were made into small pieces and placed in a medium flask. Three extraction solutions with different chloroform:methanol mixtures of ratios 2:1, 1:1, and 1:2 v/v were used. For each extraction step, the solution with SC sample was kept at room temperature under gentle shaking for about 2 hours. The SC material was collected by filtration after each step. The whole extraction sequence was repeated once again with all three solvent mixtures for about 30 minutes for each extraction time. The filtered SC material was soaked in methanol overnight. In the final step, the methanol extracted corneocyte materials were rinsed in MilliQ several times and dried under vacuum in a desiccator.

### Sample preparation

Samples for X-ray diffraction studies were hydrated as follows; small pieces (∼5–10 mg) of dry SC or corneocytes were placed on a pan in the DVS (Dynamic vapor sorption, Surface Measurement Systems Ltd., London, UK) sorption microbalance with a stream of nitrogen to control the RH. The samples were hydrated for about 24–36 h to reach stable conditions (when the rate of weight change is <10^−4%^/min) at 32 °C and the desired RH as controlled in the sorption microbalance. The urea-containing samples were prepared as follows: ∼5 mg of dry SC or corneocytes powder was mixed with a small volume of aqueous solution containing the desired amount of urea (adjusted to give sample composition of 20 or 30 wt% urea with respect to the dry weight of SC or corneocyte samples). The mixing was followed by vigorous vortexing. The excess water was then evaporated in a desiccator under vacuum. The samples were subsequently placed in 80% RH chamber at 32 °C for 48 h to reach hydration equilibrium conditions. The SC and corneocyte samples were then transferred (within one minute) into the screw-tight sandwich cells with polyethylene films in-between to avoid dehydration. The samples were subsequently mounted on a sample holder in the X-ray device for measurements.

For NMR experiments, the SC and corneocyte samples were made into scaly powder by the aid of mortar and pestle. In previous studies, it was shown that there are no detectable differences in terms of molecular mobility in SC lipid and protein components between the SC sheet and pulverized SC observed with PT ssNMR^40^. The powder was used because the time needed to reach stable conditions (assumed equilibrium) is faster compared to SC sheets. Approximately 25 mg of dry corneocytes or SC sample was used for NMR sample preparation. For hydration at different RH, the samples were placed either in a DVS sorption microbalance with a nitrogen stream at controlled RH, or in a desiccator with a saturated salt solution to maintain the desired RH, e.g. KNO_3_ for 90% RH. The temperature was set to 32 °C in the sorption microbalance and desiccator. The samples were hydrated for about 24–36 h to reach stable condition and then transferred quickly in a minute into the NMR inserts (Bruker) for measurements.

For sorption measurements, ∼5–8 mg dry powder of SC and corneocyte samples was used in the DVS sorption pan. The FTIR experiments was also performed with ∼1–2 mg of powder samples and to measure in the fully hydrated condition, the powder samples were hydrated in D_2_O for about 24 h at 32 °C prior measurements.

### X-ray diffraction

WAXD studies were performed using in-house X-ray setup, GANESHA 300 XL SAXS system (JJ-Xray, Denmark). The scattering intensity (*I*) was measured as a function of scattering vector *q* (in reciprocal Ångström). The latter is defined as $q=\frac{4\pi \sin \theta }{\lambda }$, where *θ* is the scattering angle and *λ* is the wavelength of the incident beam, which is 1.54 Å in this case. The sample to detector distance was adjusted based on the *q* range selected. From the different position of *q*, the *d*– spacing was calculated using the equation $d=\frac{2\pi }{q}$. Diffraction data were collected on a PILATUS 2D photon counting detector (Dectris, Switzerland). The scattering data were collected for about 20 minutes at 32 °C.

### Solid state NMR

The NMR method comprise three types of measurements: DP (direct polarization), CP (cross polarization)^41^ and INEPT (insensitive nuclei enhanced by polarization transfer)^42^. All NMR experiments were performed on a Bruker Avance AVII 500 NMR spectrometer that is equipped with a Bruker E-free 4 mm MAS (magic angle spinning) probe. The operating frequency of MAS is 5 kHz and the ^1^H and ^13^C resonance frequencies were 500 and 125 MHz, respectively. The temperature was chosen to be 32 °C to maintain physiological skin temperature and was calibrated by using methanol^43^. All NMR spectra were recorded under 68 kHz two-pulse phase modulation (TPPM) ^1^H decoupling^44^ and with a spectral width of 250 ppm. ^1^H and ^13^C hard pulses were given at *ω*_1_^H/C^/2π = 80 kHz. For CP experiments, the ^13^C nutation frequency was 80 kHz and the ^1^H nutation frequency linearly ramped from 72 to 88 kHz during 1 ms contact time. For INEPT, the delay times of *τ* = 1.8 ms and *τ*′ = 1.2 ms were used. A total of 2048 scans were recorded per experiment with an acquisition time and recycle delay of 0.05 and 5 s, respectively. This gives a total experimental time of ∼9 h for all three sets of measurements (DP, CP and INEPT) for a given sample. The ^13^C chemical shift scale was externally referenced to the methylene signal of solid α-glycine at 43.7 ppm. The experimental time domain data was processed with line broadening of 20 Hz, zero filling from 1597 to 8192 time domain points, Fourier transformation, phase correction^45^ and baseline correction by using in-house Matlab code partially derived from matNMR^46^.

### Sorption microbalance measurements

Sorption measurements were recorded for intact SC and isolated corneocytes using DVS sorption microbalance. To compare the sorption isotherm, SC and corneocyte samples were run at the same time in a single experiment. The dry samples were placed on two pans in the DVS microbalance and exposed to a stream of nitrogen with controlled RH. The sorption was continuously recorded by weighing the microbalance. The equilibrium at each RH was defined by the conditions where the rate of change in mass is less than 10^−4%^/min. The sorption data are presented in terms of water content as a function of RH. The water content (wt%) is calculated as (*m*_*s*_ *− m*_s,dry_)/ *m*_*s*_, where *m*_*s*_ is the total mass of the sample including water at a given time point and *m*_s,dry_ is the weight of the dry sample at 0% RH. The differences observed in the sorption data are also reproduced. All the sorption measurements were performed at 32 °C.

### Attenuated total reflection FTIR (ATR-FTIR)

The ATR-FTIR setup consists of PerkinElmer instrument equipped with deuterated triglycine sulphate (DTGS) detector. The FTIR data collection and reduction procedure was performed using the built-in software. A total of 128 scans was acquired for each spectrum. The FTIR spectral resolution was set to 4 cm^−1^. All the spectra were collected at 25 °C.

### Data Availability

The datasets generated during and/or analyzed during the current study are available from the corresponding authors on reasonable request.

## Results and Discussion

In the present study, we aim at the characterization of hydration-induced changes on molecular organization and dynamics within the keratin filaments inside the corneocytes of SC at varying hydration conditions. We also investigate the effects of adding urea to slightly dehydrated SC and corneocyte samples. We monitor structure and dynamics in the extracellular SC lipids in the very same samples. The molecular changes are then correlated to macroscopic changes in terms of SC water swelling. We have investigated systems of intact SC and isolated corneocytes at varying water activity (*a*_*w*_), as controlled by the RH of the sample surrounding (*RH* = *a*_*w*_ × *100%*). The WAXD measurements provide information on the molecular organization of small structural unit in both SC lipids and keratin filament components. The ATR-FTIR results give insight regarding protein secondary structure and hydrocarbon ordering, supporting the WAXD data. The PT ssNMR method is sensitive to the presence of a small amount of mobile (fluid) fraction in the complex SC material, and it provides information on molecular dynamics in different SC components with close to atomic resolution. Sorption measurements were performed to monitor the water uptake in SC and isolated corneocytes at varying RH. From the combination of experimental studies, we obtain a detailed picture that makes it possible to link between molecular properties in SC components, and macroscopic properties in terms of SC water-holding capacity.

### Hydration effects on keratin filament structure

WAXD is a powerful tool to probe short range structural ordering (∼3–15 Å) in the high *q* range, and it can provide information on the secondary structure and higher organization of proteins in the keratin filaments as well as packing of the lipid hydrocarbon chains. The WAXD peak assignment is based on the comparison with previously published X-ray data on SC systems at fixed water content^47–50^.

**Figure 2:**
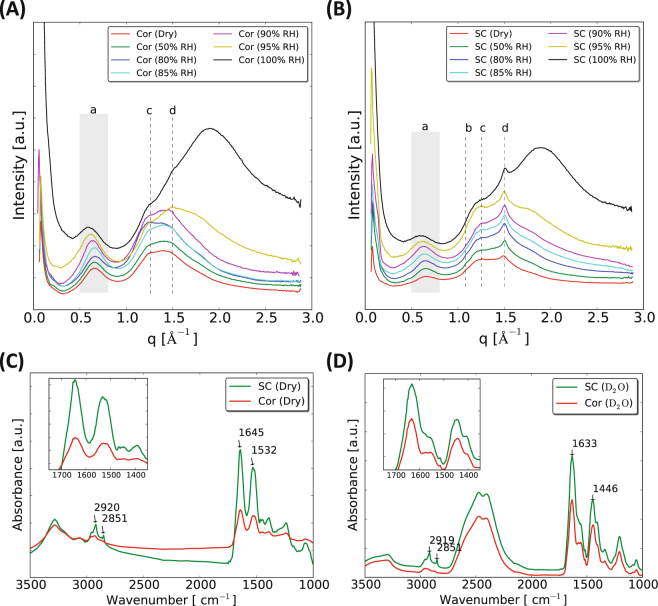
WAXD pattern of isolated corneocytes (**A**) and intact SC (**B**) hydrated and measured at 32 °C and at varying RH. The different colors in the spectra indicate the different hydration levels as controlled by the RH in the sample surrounding. The peaks in the shaded area of the WAXD spectra (a – keratin interchain distance) indicate a change in *q* position with respect to the hydration whereas the peaks marked in dashed lines in the spectra (b – *α*-helix, c – *β*-sheet & d – lipid chain packing) do not show any change with hydration. FTIR spectra (1000–3500 cm^−1^ region) of SC (green) and isolated corneocytes (red) in dry (**C**) and hydrated condition (**D**) measured at 25 °C. The broad peak around 2500 cm^−1^ and a sharp peak at ∼1200 cm^−1^ in panel D are due to the contribution from D_2_O hydration. Inset shows zoomed view of bands originating from protein secondary structure in the spectral region 1400–1700 cm^−1^. Corneocytes abbreviated as Cor in the legends.

Figure 2A and B show the WAXD spectra obtained from the isolated corneocytes and intact SC equilibrated at different RH. All experiments were performed at 32 °C, which represents physiological skin temperature. Selected samples were also studied at ambient room temperature of 25 °C. The WAXD data in Fig. 2A show that the dry corneocytes give rise to a prominent peak at *q* = 0.66 Å^−1^, which corresponds to a *d*– spacing of 9.5 Å (Fig. 2A, grey shaded area - a; red curve). This peak was previously observed for intact SC, and has been attributed to the interchain distance between two α- helical polypeptides intertwined together in a coiled coil dimer within the keratin filament (Fig. 1)^47–49^. The corresponding peak is also detected in the spectra obtained from the dry SC sample (Fig. 2B, grey shaded area - a; red curve) at a *q* position of ∼0.65 Å^−1^, corresponding to *d*– spacing of ∼9.6 Å. When the water activity in the SC and corneocyte samples increase, this peak becomes broader. For water activities above 0.85, the position of this peak shifts towards lower *q* values, indicating an increase of the interchain distance. For the fully hydrated samples, the WAXD spectra indicate an interchain distance of ∼10.7 and 10.6 Å, for intact SC and isolated corneocytes, respectively (Fig. 2A and B, black curves). Similar trends in interchain distance with varying RH were also observed at the lower temperature at 25 °C (supplemental Fig. S1A and B). At 100% RH, a broad peak at ∼1.9 Å^−1^ is evident, which is due to the water vapor in the sample cell as shown in the supplemental Fig. S1C.

Information about protein secondary structures can be obtained from the WAXD and FTIR data. The WAXD spectra from intact SC reveal a resolved peak at *q* = ∼1.28 Å^−1^ (Fig. 2B, c – dashed line), which corresponds to a *d*– spacing of ∼4.9 Å. This peak arises from the secondary *β*-sheet configuration in the keratin proteins^50^. A *β*-sheet peak with similar *d*– spacing is also observed in other systems of aggregated protein, for example, amyloid fibrils^51^. In the corresponding spectra from the isolated corneocytes, this peak cannot be resolved for RH < 90% due to the presence of overlapping broad peaks in this spectral regime (Fig. 2A, c – dashed line). The position of the *β*-sheet peak does not change with the variation in hydration, although there is an indication of increase in the peak intensity with increasing RH. In the spectra from intact SC at RH < 85%, there is also evident a small peak at *q* = ∼1.1 Å^−1^, apparent as a small shoulder on the large peak (Fig. 2B, b – dashed line). This peak corresponds to a *d*– spacing of ∼5.4 Å, which might be due to the presence of secondary *α*-helical conformation in the proteins^50^. Again, there is no change in the *α*-helix peak position with the variation in RH, although, there is an indication of gradual decrease in peak intensity at higher hydration levels. The peak corresponding to *α*-helical structure cannot be resolved in the spectra from the isolated corneocytes (Fig. 2A). The secondary *α*-helix and *β*-sheet structures are also detected for the corneocytes and SC samples at 25 °C (supplemental Figs S1A and S2B).

The interpretations regarding protein secondary structures are further supported by the FTIR spectra of SC and isolated corneocytes in the dry and fully hydrated condition shown in Fig. 2C and D. For technical instrument reasons, the experiments were only performed at 25 °C, and the data are compared to WAXD data at 25 °C and 32 °C. The spectral bands located in the region 1400–1700 cm^−1^ are prevailed by protein absorption. The amide I component (C=O) in the spectra of SC and isolated corneocytes in the dry state (Fig. 2C) is observed at ∼1645 cm^−1^. This value is typical for the *α*-helical secondary structure in the keratin filaments^52^. Upon hydration, a redistribution of amide I band frequency from ∼1645 to ∼1633 cm^−1^ in conjunction with a weak shoulder at ∼1565 cm^−1^ is observed in both the spectra from intact SC and isolated corneocytes (Fig. 2D). This may indicate a transition to the secondary *β*-sheet structure with hydration^52–54^, which is also consistent with the observed changes in the peak intensities in the WAXD spectra (Fig. 2A and B). The amide II (N-H) maximum band intensity in the spectra obtained from intact SC and isolated corneocytes is detected at ∼1532 cm^−1^ (Fig. 2C), which is somewhat lower compared to the typical band frequency of ∼1550 cm^−1^, reported previously^54^. The FTIR data suggests the presence of *β*- sheet structure in the corneocytes protein in the dry state, thus supporting the interpretation of the WAXD data (Fig. 2B). When the samples were hydrated in D_2_O, the amide II band frequency shifts to a lower wavenumber at ∼1446 cm^−1^ due to H-D exchange (Fig. 2D). A shoulder at ∼1406 cm^−1^ is also evident, which again suggests the formation of *β*-sheet structure^53^.

Finally, the WAXD and FTIR spectra also contain information on the SC extracellular lipids. In the dry SC, a distinct peak is observed at *q* = ∼1.5 Å^−1^ (Fig. 2B, d – dashed line; red curve), corresponding to a distance of ∼4.1 Å. This peak is indicative of a hexagonal lateral packing of lipid hydrocarbon chains in the solid lamellar lipid phase^49^. No additional peak at ∼3.7 Å is observed, which indicates the absence of orthorhombic lateral packing of the lipid hydrocarbon chains, as previously observed for human SC but not porcine SC^49,55,56^. The position of the lipid peak remains constant at varying RH. This peak confirms that the crystalline packing of the lipids is not altered by hydration in any detectable way. This peak is also observed in the SC at 25 °C (Supplemental Fig. S1B). In the WAXD spectra of the isolated corneocytes (Fig. 2A), a small lipid peak is detected at RH > 90% (Fig. 2A, d – dashed line). The intensity of the peak originating from the lipid hydrocarbon chains is much lower in the spectra from the corneocyte samples as compared to the spectra from the intact SC, confirming that the majority of the lipids were removed in the extraction procedure when isolating the corneocytes. A small peak detected from the lipids in the spectra of isolated corneocytes may indicate that the remaining lipids in the corneocyte envelope exhibit hexagonal packing^49^, or that the extraction was not fully complete and there is some residual unbound extracellular SC lipids present in the sample^48^. Again, the results obtained from WAXD (Fig. 2A and B and supplemental Fig. S1A and B) can be compared with the FTIR measurements (Fig. 2C and D). The spectral band located in the region 2800 and 3000 cm^−1^ is characteristic for CH_2_ stretching vibrations of hydrocarbon chains. The hydrocarbon CH_2_ symmetric and asymmetric stretching band frequencies in the SC are observed at 2851 and 2920 cm^−1^ (Fig. 2C and D, green)^54,57^. The intensity of these bands is drastically reduced in the corresponding corneocytes spectra (Fig. 2C and D, red).

**Figure 3:**
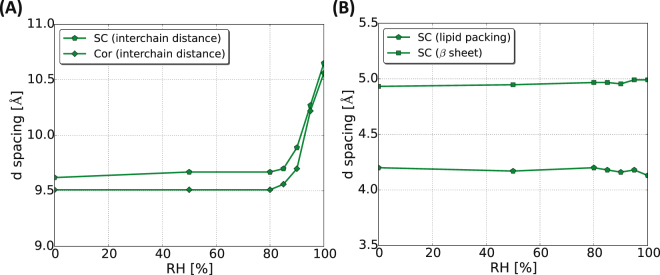
The *d*– spacing values of keratin interchain distance for (**A**), lipid chain packing and protein secondary structure (**B**) are presented as a function of RH for both corneocytes and SC samples. The *d* values were calculated from the peak positions in *q* in Fig. 2.

To summarize the results obtained from the WAXD experiments, Fig. 3A and B illustrate how the *d*– spacing (calculated from the individual peak position in *q*) for the most prominent peaks are vary as a function of RH. From this we conclude that the keratin interchain distance is sensitive to changes in hydration at RH > 85%, while distance of the peak originating from the lateral packing of SC lipids as well as the β-sheet structure in the keratin filaments is unaffected by the variations in RH. The same behavior is shown both for isolated corneocytes and intact SC. To further probe the nature of the observed abrupt change in keratin filament structure around 85% RH, we investigated molecular dynamics in different amino acid segments in the keratin filaments by means of PT ssNMR.

### Molecular mobility in keratin filaments as revealed by PT ssNMR

The effect of hydration on the molecular dynamics in different amino acid segments in the keratin filaments was investigated using PT ssNMR on natural abundance ^13^C. A detailed description of the method is given in ref.^58^. In previous studies, the method was used on intact SC, showing that all amino acids in the keratin filaments are rigid in dry conditions, while the Ser and Gly residues in the terminal segments of the keratin protein become mobile at higher RH^24^. Here we systematically investigate this rigid-mobile transition at varying RH in samples composed of either isolated corneocytes or intact SC. The PT ssNMR method comprises DP, CP and INEPT experiments. The DP is used as a reference spectrum as it presents resonances from all carbons in the sample. The CP and INEPT experiments involve transfer of polarization from ^1^H nuclei to neighboring ^13^C nuclei, although they rely on different mechanisms of transfer. The CP scheme involves polarization transfer through space via dipolar couplings and is normally used to enhance signal in the solid state NMR^41^. For INEPT, the polarization transfer occurs through covalent bonds via scalar couplings and is efficient in enhancing signals in liquid state NMR^42^. The magnitude of CP and INEPT signal for a particular segment can vary depending on the rotational correlation time (*τ*_c_) and the ^13^C−^1^H bond order parameter (*S*_CH_). The *S*_CH_ and *τ*_c_ quantifies anisotropy and the rate of C-H motion. A schematic model of how the CP and INEPT signal depends on *τ*_c_ and *S*_CH_ is presented in supplemental Fig. S2. As explained in detail in ref.^58^, CP is selectively enhancing signals for slow or anisotropic segments while INEPT is doing so for fast and isotropic segments. The CP signal is inefficient for fast isotropic reorientation whereas INEPT lacks the signal for slow motion. Comparing the signals of CP and INEPT with respect to the DP signals, atomically resolved information on the structure and dynamics of different molecular segments of the sample can be obtained, which can be described in terms of mobility and rigidity. We can define a molecular segment as ‘rigid’ when only a CP signal is detected whereas a signal from INEPT could define a molecular segment as ‘mobile’. Due to the non-linear response of CP and INEPT signals with respect to changes in molecular motions and anisotropy (Fig S2), the PT ssNMR method cannot be used for direct quantification of the mobile to solid ^13^C fractions for certain segments^58^.

**Figure 4:**
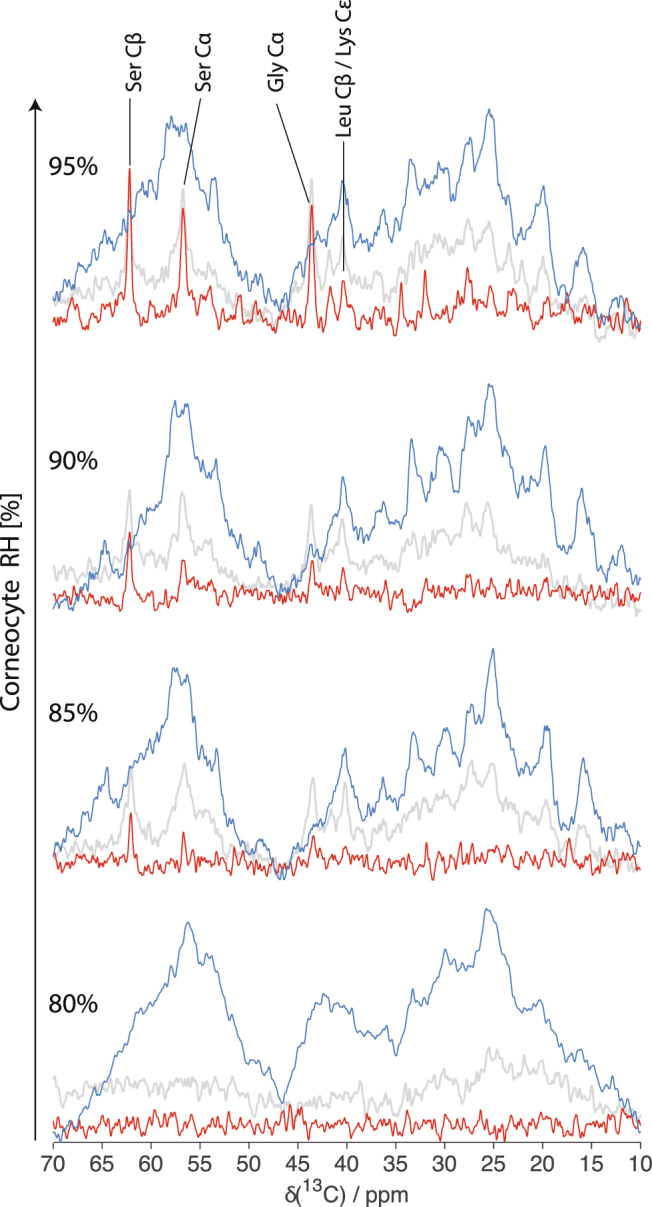
PT ssNMR ^13^C MAS spectra (DP; grey, CP; blue and INEPT; red) of corneocyte samples hydrated at different RH and measured at 32 °C. The resonance lines from Ser and Gly amino acid residues in the protruding keratin terminal chains are visible in the INEPT spectra. The INEPT intensity from these residues increases with increasing RH.

The PT ssNMR spectra from corneocytes at varying RH are shown in Fig. 4. The prevailing broad CP signal (blue) exist in all the spectra implies that the majority of all carbon molecular segments present in the samples are rigid under all conditions investigated (Fig. 4). At 80% RH, the lack of INEPT (red) signal implies that there is no trace of mobile carbons in any amino acids (Fig. 4). The NMR spectra obtained at 80% RH indeed look similar to that of dry corneocytes (Supplemental Fig. S3A), and the absence of INEPT signal indicates a fully rigid structure. At 85% RH, a small INEPT signal is detected at chemical shifts corresponding to the amino acids serine (Ser C_α_ and Ser C_β_ at ∼57 and 62 ppm, respectively) and glycine (Gly C_α_ at ∼44 ppm) (Fig. 4). These amino acid residues are abundantly present in the protruding terminals of keratin filaments^24^. The mobility in these segments gradually increased when the RH is increased further. At 90% RH, additional mobility was also observed for Leu C_β_ and/or Lys C_ε_ (∼41 ppm). The Leu and Lys amino acid residues are highly enriched in the coiled coil core of keratin filaments^24^. Similar behavior was also observed for the amino acids Ser, Gly, Leu and Lys in the keratin in samples of intact SC at varying RH (Supplemental Fig. S3B and ref.^24^). The samples of dry intact SC contain a minute fraction of mobile segments found in the terminal of lipid acyl chains (ωCH_3_ at ∼14.6 ppm and (ω − 1)CH_2_ at ∼23.3 ppm) (Supplemental Fig. S3B), and this fraction of mobile lipids gradually increases with increasing RH^24^.

### Urea can preserve hydrated structures at reduced RH

**Figure 5:**
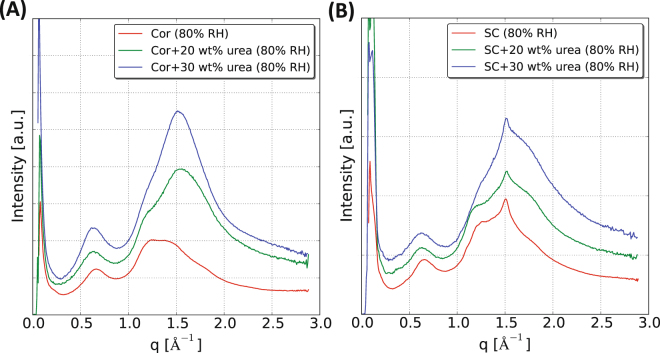
WAXD pattern of corneocytes (**A**) and SC (**B**) hydrated at 80% RH with the addition of 20 and 30 wt% urea. The different spectra are color-coded as described in the figure legend. All the measurements were performed at 32 °C.

To treat dry skin conditions, it is common to apply a skin care product containing small polar molecules named humectants, for example, urea or glycerol. We here investigate the effect of urea on the corneocytes and SC at conditions of 80% RH (Fig. 5A and B). The addition of 20 wt% urea to the isolated corneocytes leads to a shift of the peak corresponding to the keratin interchain distance from *q* = 0.65 Å^−1^ in the urea-free sample to *q* = 0.62 Å^−1^ in the sample with 20 wt% urea, which corresponds to a change in *d*– spacings from 9.5 to 10.1 Å (Fig. 5A, green curve). A similar shift from 9.6 to 10.2 Å was observed for the samples of intact SC at corresponding conditions (Fig. 5B). Increasing the urea concentration further to 30 wt% does not lead to any further increase in the keratin interchain distance, although the intensity from the protein secondary structure appear reduced at the higher urea concentration (Fig. 5A and B). The lipid chain packing in the SC (Fig. 5B) is not affected by the addition of urea as the peak position at ∼1.5 Å^−1^ remains unchanged in all the spectra. The results can be related to previous findings of the same systems using PT ssNMR, showing that the addition of 20 wt% urea to isolated SC or isolated corneocytes at 80% RH gives rise to similar NMR spectra as the SC or corneocytes without added compound at 96% RH^37^. In other words, both interchain distance and molecular mobility in the terminal segments of the keratin filaments respond in a similar way to the increase in RH and to the addition of urea at constant (reduced) RH. The observed effects can be explained by the fact that polar humectant compounds, like urea, have low vapour pressure and therefore remain in the SC also at reduced RH when water evaporates. In this way, urea can substitute for water under dehydration in such a way that the properties of the system remain largely unchanged as compared to a more hydrated state^37^. This is an important role of NMF in the SC, and it can be related to the effects of osmolytes in other biological systems under osmotic stress.

### Relation between water uptake, structure and molecular mobility

**Figure 6:**
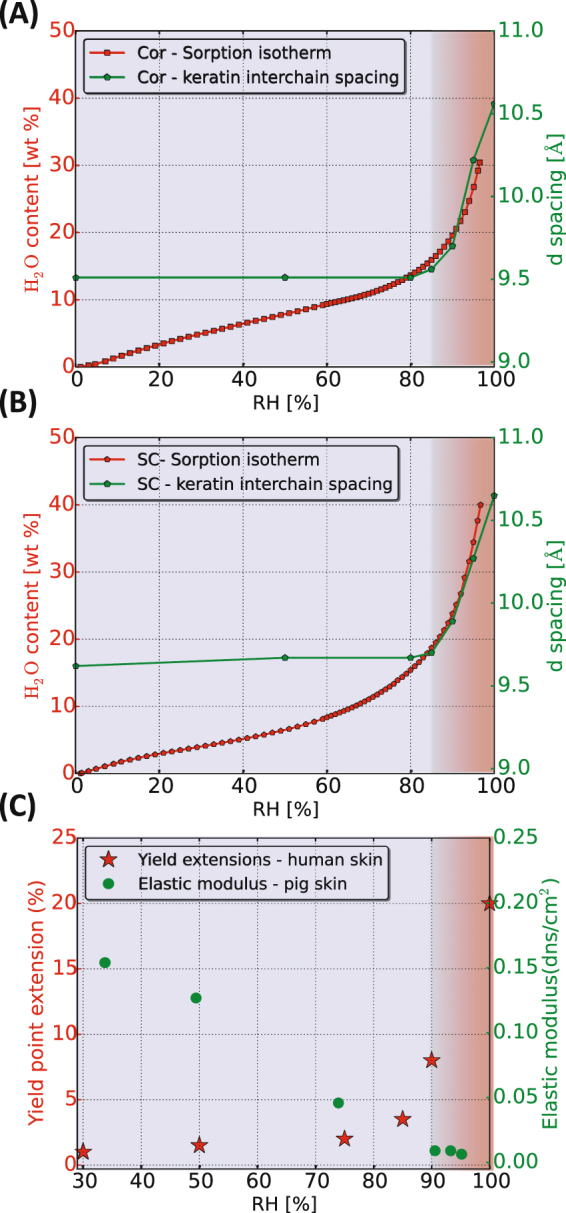
Sorption measurements performed at 32 °C expressed in water contents, wt% (left y-axis) and keratin *d*– spacing (right y-axis) are plotted as a function of RH for corneocytes (**A**) and SC (**B**). The blue shaded area indicates the presence of CP signals from rigid segments, which is detected for all RH values. The red shaded area indicates that INEPT signal was detected for Ser and Gly amino acid residues of keratin, indicating mobility in these segments. The gradient in the red shading implies the gradual change of the INEPT signal corresponding to increasing molecular mobility. (**C**) Elastic modulus (right y axis) and yield point extensions (left y axis) values were calculated at various RH’s for porcine and human skin based on the literature data presented in refs^31^ and^36^.

The observed changes in molecular properties of the keratin filaments with variations in RH have impact on the macroscopic properties of the corneocytes, and it may influence the material properties and water-holding capacity of the intact SC. Here, we aim to correlate the molecular changes described above to water uptake in corneocytes and intact SC at varying RH. The water sorption isotherms of isolated corneocytes and intact SC are shown in Fig. 6A and B (red curves). The isotherms provide a relation between the water content (expressed in terms of wt% relative to the dry sample) and the RH of the surrounding vapor phase. The sorption isotherms (Fig. 6A and B) show a small and gradual uptake of water over a large range of RH ranging from 0 to ca. 85%. At higher RH, the sorption isotherms become steeper, which indicates that a small change in RH leads to a large water uptake in the samples. The sorption isotherm of intact SC closely resembles that of the isolated corneocytes, although the water uptake is slightly higher in the sample of intact SC at higher RH.

In Fig. 6A and B, the sorption isotherms are plotted together with the data for keratin interchain *d*– spacings obtained from the WAXD measurements (green curves). The color code in the figures illustrates the changes in the molecular mobility in Ser and Gly residues obtained from PT ssNMR at varying RH where blue signifies the absence of INEPT signal, and the gradual change in red color illustrates increased molecular mobility in these segments. When comparing these sorption isotherm, keratin *d*– spacing and PT ssNMR data in Fig. 6A and B, it is noted that the onset of the high water uptake coincides with the RH where we detect an increase in the interchain distance within the keratin filament as well as induced mobility in the protruding terminal segments of the keratin filaments. When comparing with literature data, the elastic properties of porcine and human SC at varying RH (Fig. 6C) also show response to the variation in RH, with abrupt changes in properties around between 75 and 90% RH^31,36^. Taken together, these observations illustrate a threshold hydration level associated with molecular changes in terms of structure and molecular dynamics in the keratin filament. The observed molecular changes coincide with changes in SC mechanical properties as well. The threshold limit lies around 85% RH for both intact SC and isolated corneocytes. The hydration-induced brittle to ductile transition in SC has previously been associated with the hydration-induced glass transition in the keratin molecules^34^. To our knowledge, there is no literature study report the precise determination of how the glass transition temperature varies with hydration for SC keratin. However, it has been demonstrated for both hair and wool keratin that upon hydration the glass transition temperature reduced dramatically^59,60^, implying the possibility of hydration-induced transition at physiological temperatures. In general, an increase in water activity can lead to phase transitions analogous to temperature-induced transitions for many self-assembled systems composed of, for example, proteins, lipids and surfactants^58,61–64^.

The addition of urea at slightly dehydrated conditions has also been shown to affect the macroscopic properties of SC. It has previously been reported that urea influences the water uptake in SC^31,37^. Indeed, the total content of polar solvent in SC sample treated with urea at 80% RH was shown to be close to the water content in SC at 97% RH^65^, again showing similar response in SC properties to increased hydration by variation in RH and the addition of urea at constant (reduced) RH. The threshold RH where SC was characterized by low elastic modulus also shift to lower RH in the presence of urea^31^. Taken together, these examples illustrate that the addition of urea to SC at reduced RH leads to similar molecular and macroscopic responses as the increase in RH for SC without urea. These findings provide new molecular insight into how small polar molecules in NMF and skin care formulations act to protect the skin from drying. In most practical situations, the distribution of water, NMF and added molecules in SC cannot be considered uniform^66,67^. The upper layer of the skin generally has the lowest water activity, while the deeper layers are close to physiological water activity (corresponding to 99.6% RH).

**Figure 7:**
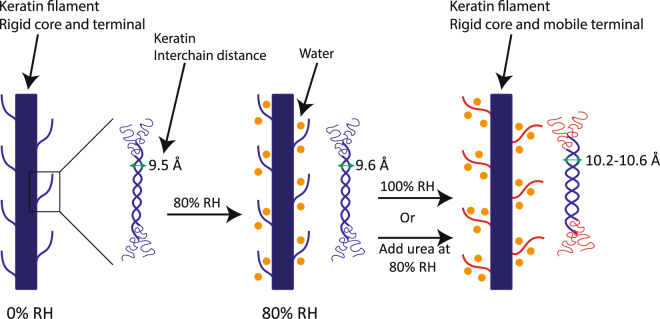
Schematic drawings of a keratin filament (left) and a zoomed portion of keratin filament showing coiled coil dimer present within the core of keratin filament (right) at varying RH. The blue keratin core with terminals attached to the coiled coil indicates solid form of all amino acid segments at both dry conditions and at 80% RH. The red terminals attached to the coiled coil of the keratin core signify mobility in the protruding terminals at 100% RH or upon the addition of urea at constant RH (here at 80% RH). The arrow in the coiled coil dimer indicates the interchain distance between two *α*-helical polypeptide chains (see Fig. 1), showing that hydration leads to an increase in the interchain distance when going from the dry state to 100% RH.

A cartoon representation of our interpretation of hydration effect on the keratin filament is presented in Fig. 7. The blue keratin core with protruding terminals in the dry state is indicative of rigid segments present in all amino acids. The keratin filament appears virtually insensitive to changes in hydration up to ca. < 85% RH. At higher RH values, the terminal segments in the protruding chains in the keratin filaments become mobile, which also influences the arrangement of the chains in the intertwined peptide segments (Fig. 7, right). The amino acids in the core of the keratin filament remain rigid at all hydration conditions. The terminal segments in the keratin filaments are protruding out from the solid rod-like structure. The terminal segments in contact with the surrounding solvent are the part of the keratin filament that is most easily affected by the changes in hydration or the addition of humectants. The hydration-induced mobile protruding segments on the rigid keratin filaments can be compared to flexible polymer brushes used to stabilize, for example, colloids^68^. When the polymer brushes on different particles approach each other, there is a reduction in the configurational freedom of the flexible chains, which generates a repulsive force of entropic origin^69^. In the dry state at lower RH values, the terminal chains in the keratin filament are rigid, forming a collapsed structure, and the repulsive force between the keratin rods is relatively short-ranged. Consequently, there is only moderate swelling of the keratin-filled corneocytes in these conditions. At higher RH values or in the presence of urea, there is a conformational change in the terminal segments to form more flexible and mobile structures– similar to extended polymer brushes- which leads to more long-ranged repulsion and swelling of the corneocytes. This conformational change is also consistent with recent modeling of interactions between keratin filament in the presence of ions or NMF compounds^70^. The structural changes in keratin can explain present observations that interchain distance and water uptake increase at conditions close to the threshold RH where the terminal segments become mobile. The analogy to polymer brushes at intermediate keratin filaments has previously also been made for the flanking terminal regions in neurofilament structural assembly^71,72^. The present study can therefore also provide novel insights to the more general problem of how intermediate filament organizes in response to variations in surrounding conditions^73^.

## Conclusions

In the present study, we investigate the interplay between molecular and macroscopic properties of SC and how this varies with hydration conditions. We also examine the effect of NMF compound urea on the SC molecular changes at reduced hydration condition. We use complementary experimental techniques to characterize molecular organization and dynamics as well as water uptake in intact SC and isolated corneocytes. The main conclusions are:

- At the molecular level, there is correlation between changes in the interchain distance of keratin intermediate filaments and the molecular dynamics in the Gly and Ser amino acid residues in its protruding terminal segments. There is response in both structure and dynamics at RH’s humidities above the threshold limit of 85% RH, while no detectable changes are shown for lower RH’s. The same conclusions are drawn for intact SC and isolated corneocytes.
- At the macroscopic level, we detect a response in water uptake to variations in RH for RH > 85%, while the swelling profile is rather shallow at lower RH’s. Comparisons with previous studies of SC mechanical properties also suggest abrupt changes in SC material properties around similar threshold RH’s.
- There is a correlation between the molecular and macroscopic properties with respect to hydration, where the abrupt changes in SC molecular organization and dynamics coincide with changes in SC macroscopic swelling properties. The flexible terminals at the solid keratin filaments can be compared to flexible polymer brushes, creating long-range repulsion and extensive swelling in water.
- The addition of the NMF compound urea to SC at reduced RH lead to similar molecular and macroscopic responses as the increase in RH for SC without urea. The same conclusions are drawn for intact SC and isolated corneocytes. These findings provide new molecular insight into how small polar molecules in NMF and skin care formulations act to protect the skin from drying.

## Electronic supplementary material

Supporting information
