# Supplementary material for: Skin hydration: interplay between molecular dynamics, structure and water uptake in the stratum corneum
Source: Sci Rep. 2017 Nov 16;7:15712. doi: 10.1038/s41598-017-15921-5 (PMC5691061; doi:10.1038/s41598-017-15921-5)
Supplement: Supplementary file 1 — Supporting information [file 41598_2017_15921_MOESM1_ESM.pdf]

Skin hydration: interplay between molecular dynamics, structure and water uptake in the stratum corneum

Enamul Haque Mojumdar[1*], Quoc Dat Pham[1], Daniel Topgaard[1] and Emma Sparr[1*]
[1]*Division of Physical Chemistry, Center for Chemistry and Chemical Engineering, Lund University, P.O. Box 124, SE-22100 Lund, Sweden*

Corresponding authors
Enamul Haque Mojumdar – enamul.mojumdar@gmail.com;
Emma Sparr – emma.sparr@fkem1.lu.se;

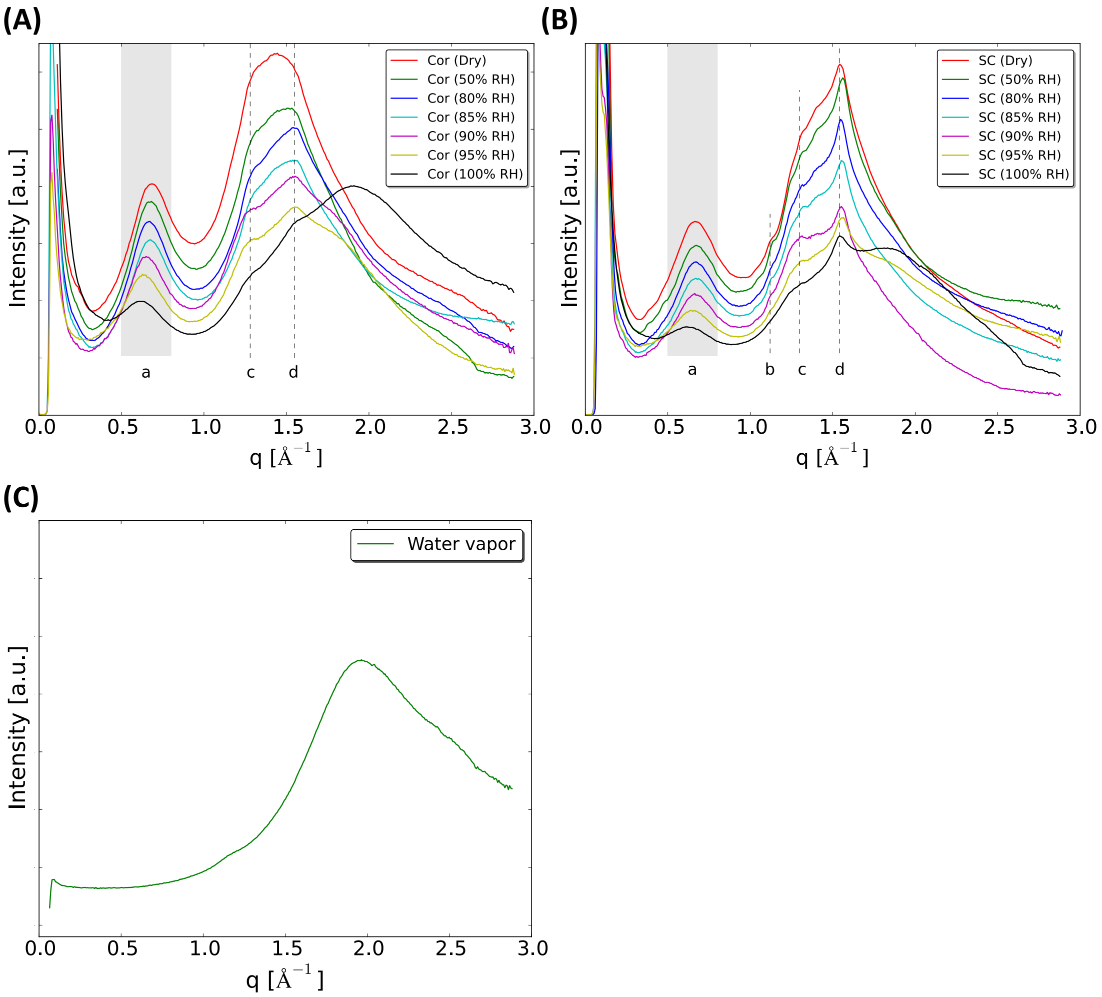

Figure S1. WAXD spectra of corneocytes (A) and intact SC (B) hydrated and measured at 25 °C. The different hydration levels controlled by the RH in the sample surrounding are color coded in the spectra. WAXD pattern of water vapor measured at 32 °C (C). The spectrum shows a broad peak at ~1.9 Å^{-1}, which is prevailed in the spectra of corneocyte and SC at 100% RH at 25 and 32 °C.

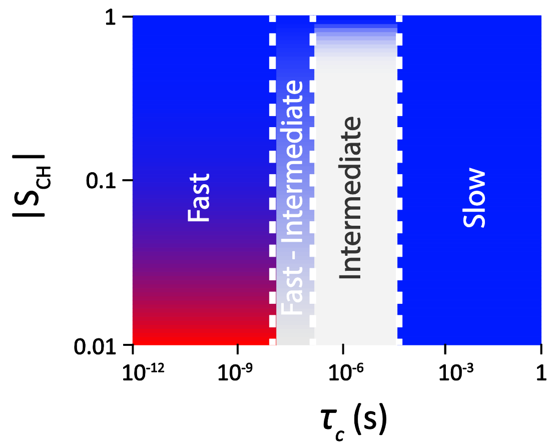

Figure S2. Theoretical model of INEPT (red) and CP (blue) efficiency as a function of the correlation time τ_c and order parameter S_{CH} of C-H bond reorientation. The calculations were performed for a CH_2 segment at 125 MHz ^{13}C resonance frequency, 5 kHz magic angle spinning frequency, and pulse sequence timing parameters and RF power levels as reported in the materials and methods section of the main text. The different dynamic regimes in the model are presented as a colored maps where white indicates lack of signal for both CP and INEPT. Figure adapted from Nowacka et al[1].

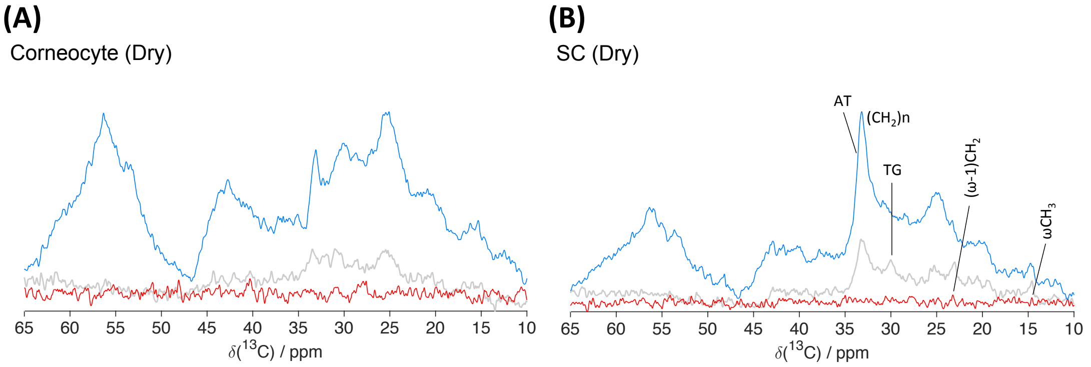

Figure S3. ^{13}C MAS spectra (DP; grey, CP; blue and INEPT; red) of dry corneocyte (A) and intact SC (B) measured at 32 °C. The dominant CP signal and lack of INEPT is indicative of the presence of only solid material in dry state at ambient temperature. In the SC, only very minor mobility in the lipid segments is visible in the dry state, which is in accordance with previous data[2]. The signature lipid peaks ωCH_3, $(\omega\text{-}1)CH_2$, all-trans (AT) and trans-gauche (TG) methylene $(CH_2)_n$ are labeled in the corresponding spectrum.

Supplementary references

1 Nowacka, A., Bongartz, N. A., Ollila, O. H. S., Nylander, T. & Topgaard, D. Signal intensities in 1H–13C CP and INEPT MAS NMR of liquid crystals. *J. Magn. Reson.* **230**, 165-175 (2013).

2 Bjorklund, S., Nowacka, A., Bouwstra, J. A., Sparr, E. & Topgaard, D. Characterization of stratum corneum molecular dynamics by natural-abundance (1)(3)C solid-state NMR. *PLoS One* **8**, e61889 (2013).
